# Metabolomic profiling in dogs with dilated cardiomyopathy eating non-traditional or traditional diets and in healthy controls

**DOI:** 10.1038/s41598-022-26322-8

**Published:** 2022-12-30

**Authors:** Caren E. Smith, Laurence D. Parnell, Chao-Qiang Lai, John E. Rush, Darcy B. Adin, José M. Ordovás, Lisa M. Freeman

**Affiliations:** 1 Nutrition and Genomics Laboratory, Jean Mayer USDA Human Nutrition Research Center on Aging, Tufts University, Boston, MA USA; 2 USDA Agricultural Research Service, Nutrition and Genomics Laboratory, Jean Mayer USDA Human Nutrition Research Center on Aging, Tufts University, Boston, MA USA; 3 Department of Clinical Sciences, Cummings School of Veterinary Medicine, Tufts University, North Grafton, MA USA; 4 Department of Large Animal Clinical Sciences, University of Florida, College of Veterinary Medicine, 2015 SW 16th Avenue, Gainesville, FL USA

**Keywords:** Biochemistry, Biomarkers, Cardiology, Molecular medicine

## Abstract

Dilated cardiomyopathy (DCM), caused by genetic and environmental factors, usually progresses to heart failure, a major cause of death in elderly people. A diet-associated form of DCM was recently identified in pet dogs eating non-traditional (NT) diets. To identify potential dietary causes, we analyzed metabolomic signatures and gene set/pathway enrichment in (1) all dogs based on disease, diet, and their interactions and (2) dogs with DCM based on diet. Metabolomic analysis was performed in 38 dogs with DCM eating NT diets (DCM-NT), 8 dogs with DCM eating traditional diets, 12 healthy controls eating NT diets, and 17 healthy controls eating traditional diets. Overall, 153 and 63 metabolites differed significantly between dogs with DCM versus healthy controls and dogs eating NT versus traditional diets, respectively, with 12 metabolites overlapping both analyses. Protein–protein interaction networks and gene set enrichment analysis identified 105 significant pathways and gene sets including aging-related pathways (e.g., nuclear factor-kappa B, oxidative damage, inflammation). Seventeen metabolites differed significantly in dogs with DCM eating NT versus traditional diets (e.g., fatty acids, amino acids, legume biomarkers), suggesting different mechanisms for primary versus diet-associated DCM. Our multifaceted metabolomic assessment of DCM in dogs highlighted diet’s role in some forms of DCM.

## Introduction

Dilated cardiomyopathy (DCM) is the second most common heart disease affecting dogs and is a leading cause of heart failure in people. In dogs, DCM most often has been considered to have a genetic basis (i.e., primary DCM, occurring in certain breeds such as the Doberman pinscher)^1^, and, in people, 30–50% of cases are attributed to genetic causes^2^. In both species, however, DCM also can develop secondary to drugs, environmental or dietary toxins, infectious agents, or nutritional deficiencies^3,4^. In light of the common progression of DCM to heart failure, which is an important cause of death in dogs and people, especially among the elderly^5–7^, recent increases in canine DCM incidence are of high interest. In 2018, the United States Food and Drug Administration (FDA) issued an alert about a possible association between diet and DCM in dogs and, to a lesser degree, cats, with two updates^8–10^. As of July 2020, more than 1100 dogs with apparent diet-associated DCM had been reported to the FDA^11^.

The cause of this current episode of secondary DCM is unknown but could be the result of a nutritional deficiency, a toxicity, or a combination of nutritional factors. Taurine deficiency, which can cause DCM in dogs and cats, appears to be uncommon in dogs with this form of DCM^12–15^. Deficiencies of other nutrients, such as thiamine, magnesium, choline, vitamin E, selenium, taurine, and L-carnitine, also have been associated with secondary DCM in humans or animal models^3,16–18^, but these nutrients do not appear to be deficient in the diets associated with this problem or in the small number of affected dogs that have been tested^15^.

The diets fed to dogs with diet-associated DCM are often "grain-free" and typically contain one or more pulses or pulse fractions (e.g., pea protein, fiber, starch) and, to a lesser degree, potatoes and sweet potatoes^12–15,19^. One study of nine diets commonly fed to dogs with diet-associated DCM and nine control diets identified four ingredients that significantly differentiated these two groups: peas and lentils (more common in the associated diets) and chicken/turkey and rice (less common in the associated diets)^20^. While standard nutritional analyses have not identified a causative factor^15^, a recent small molecules “foodomics” analysis of diets identified 122 biochemical compounds that were higher and 27 biochemical compounds that were lower in diets associated with DCM compared to those that were unassociated^20^. Random forest analysis identified the top 30 compounds that distinguished the diet groups with 100% accuracy^20^. In addition, analysis suggested that peas were the ingredient contributing to the greatest differences in biochemical compounds between the two diet groups and that peas were mostly associated with compounds that were higher in the DCM-associated diets^20^. This study provided information on biochemical compounds that could be contributing to diet-associated DCM that can be investigated further. Biochemical compounds that were higher in the associated diets are more likely to be contributing to this disease, but a smaller number of compounds that were lower also could contribute.

One obvious and paramount question is whether the biochemical compounds that distinguished the two types of diets would also be detectable in dogs eating similar associated and unassociated diets. This is one possible method of identifying the cause of diet-associated DCM. One promising approach for investigating relationships between diet and disease is metabolomics analysis, which analyzes small molecules (e.g., lipids, amino acids, drugs, plant metabolites) that can represent biomarkers of disease or diet, and can help delineate mechanism. If metabolomics analyses identify the same compounds to be higher in both DCM-associated diets and in the circulation of dogs with DCM eating these diets, it would raise the possibility that these compounds could be directly causing the disease. Alternatively, a compound in the diet might be metabolized by dogs such that the byproduct, metabolite, or set of metabolites detectable in the blood are grouped through pathway or network analyses. Conversely, a biochemical compound that was lower in the diet might be detected at lower levels in the dogs. Any metabolic changes evident from metabolomics analysis in dogs with diet-associated DCM must be differentiated from those occurring in dogs with primary DCM. Studies have shown that human patients and induced rodent models with non-ischemic DCM and congestive heart failure exhibit characteristic metabolomic signatures compared to healthy controls, although this has not been evaluated in dogs with naturally-occurring primary DCM^21–25^. Metabolomic analysis of dogs with primary and diet-associated DCM compared to healthy controls is needed to determine whether changes in metabolites and metabolic pathways in dogs with naturally-occurring DCM are similar to those in humans and induced animal models of DCM. Likewise, characterizing metabolites that are common to both diet and disease comparisons as well as those that distinguish dogs with primary DCM from those with diet-associated DCM will help to identify the cause of this current form of secondary DCM.

Therefore, the objective of this study was to use a metabolomics approach to evaluate metabolic signatures and gene set and pathway enrichment in (1) dogs based on disease (DCM or healthy), diet (non-traditional [NT] or traditional [T]), and their interactions and (2) dogs with DCM not associated with diet (presumably, dogs with primary DCM) compared to dogs with secondary, diet-associated DCM. In addition, changes before and 9 months after diet change and medical treatment were compared in dogs with diet-associated DCM. Our first hypotheses were that dogs with DCM would have different metabolic profiles compared to healthy controls and that dogs eating different diets would have different metabolic profiles. In addition, we hypothesized that metabolites and pathways that overlapped both disease and diet comparisons as well as those that differentiated dogs with diet-associated versus primary DCM could inform understanding of how diet might contribute to DCM. Finally, we hypothesized that some of the metabolic features identified in dogs with diet-associated DCM at baseline would respond to medical and dietary treatment, providing a set of potential metabolites and metabolic pathways that will be promising for further investigation.

## Results

### Study population

Plasma samples from 75 dogs at baseline were analyzed. The commercial diets eaten by the dogs were categorized as NT if they were grain-free or included pulses or potatoes in the top 10 ingredients and T if they were grain-inclusive and had no pulses or potatoes in the top 10 ingredients^10,15,26^. We assessed the following groups: Dogs with DCM eating NT diets (DCM-NT [n = 38]), dogs with DCM eating T diets (DCM-T [n = 8]), healthy control dogs eating NT diets (C-NT [n = 12]), and healthy control dogs eating T diets (C-T [n = 17]). Dogs in the four groups were not significantly different in age (*P* = 0.08), sex (*P* = 0.40), breed (*P* = 0.93), or body weight (*P* = 0.47; Table 1). The majority of dogs in both DCM groups (80% overall) had congestive heart failure present at the time of diagnosis (Table 1). After a 9-month intervention diet, 20 of the 37 dogs with DCM eating NT diets at baseline (54%) were alive and had a follow-up sample analyzed in contrast to 2 of 8 dogs with DCM eating T diets at baseline (25%; *P* = 0.47).

**Table 1:** Baseline characteristics of 75 dogs with dilated cardiomyopathy (DCM) and healthy controls eating non-traditional or traditional diets and in which metabolomic analyses were performed.

Variable	DCM (non-traditional diet)	DCM (traditional diet)	Healthy controls (non-traditional diet)	Healthy controls (traditional diet)	*P* value
n	38	8	12	17	–
Age (yrs)	7.4 (1.2–12.6)	9.2 (4.1–12.2)	5.7 (0.9–9.0)	7.7 (5.2–12.2)	0.08
**Sex**					0.40
Male	22	7	6	10	
Female	16	1	6	7	
**Breed**^ab^					0.93
Doberman pinscher	8	2	2	4	
Boxer	4	1	2	2	
Pit bull	6	0	2	1	
Mixed breed	4	0	1	3	
Golden retriever	3	1	1	2	
German shepherd	3	1	0	1	
Labrador retriever	2	1	0	2	
Great dane	2	1	1	0	
Other	6	1	3	2	
Body weight (kg)	33.3 (3.8–65.0)	38.5 (29.7–75.6)	33.1 (19.5–67.0)	33.4 (20.3–48.7)	0.47
Congestive heart failure	31 (82%)	6 (75%)	–	–	0.32

^a^Other breeds included were English bulldog (n = 2) and 1 each of Akbash, Border collie, Briard, Catahoula Cur, Coonhound, Dalmatian, Irish wolfhound, Jack Russell terrier, Saluki, and standard poodle.

^b^Dogs in the non-traditional diet group that survived to the 9-month follow-up visit included Doberman pinscher (n = 4), pit bull (n = 4), Boxer (n = 3), mixed breed (n = 3), golden retriever (n = 2), and 1 each of German shepherd, Irish wolfhound, Labrador retriever, and Standard Poodle. Dogs in the traditional diet group that survived until the 9-month follow-up visit included 1 Dalmatian and 1 Great Dane.

## Part 1: Analysis of Disease, Diet, and their Interaction in 75 dogs

### Metabolomic signatures

We first evaluated metabolites that differentiated disease status in the 75 dogs at baseline (DCM-NT + DCM-T vs. C-NT + C-T). One hundred fifty-three of 1027 metabolites assayed were significantly different based on disease status and 474 metabolites were nominally significant (Supplementary Table S1). Among the metabolites that differentiated dogs with DCM from controls were amino acids including creatine, glutamine, glutamate, and glycine; markers of glutathione metabolism; acyl carnitines (e.g., arachidoylcarnitine (C20), oleoylcarnitine (C18:1); hippurate; and tricarboxylic acid (TCA) cycle metabolites such as alpha-ketoglutarate.

We also evaluated metabolites that differentiated dogs eating different diets (DCM-NT + C-NT) versus (DCM-T + C-T). At baseline, 63 metabolites differed significantly by diet status and 229 metabolites were nominally significant (Supplementary Table S1). Among the metabolites that differentiated dogs based on diet were amino acids or amino acid-derived compounds (e.g., homoarginine, tryptophan betaine, imidazole lactate), xenobiotics (e.g., glycitein sulfate, genistein sulfate), 2-hydroxyhippurate (salicylurate), and unnamed compounds (e.g., X-25419, X-26008, X25247).

**Figure 1:**
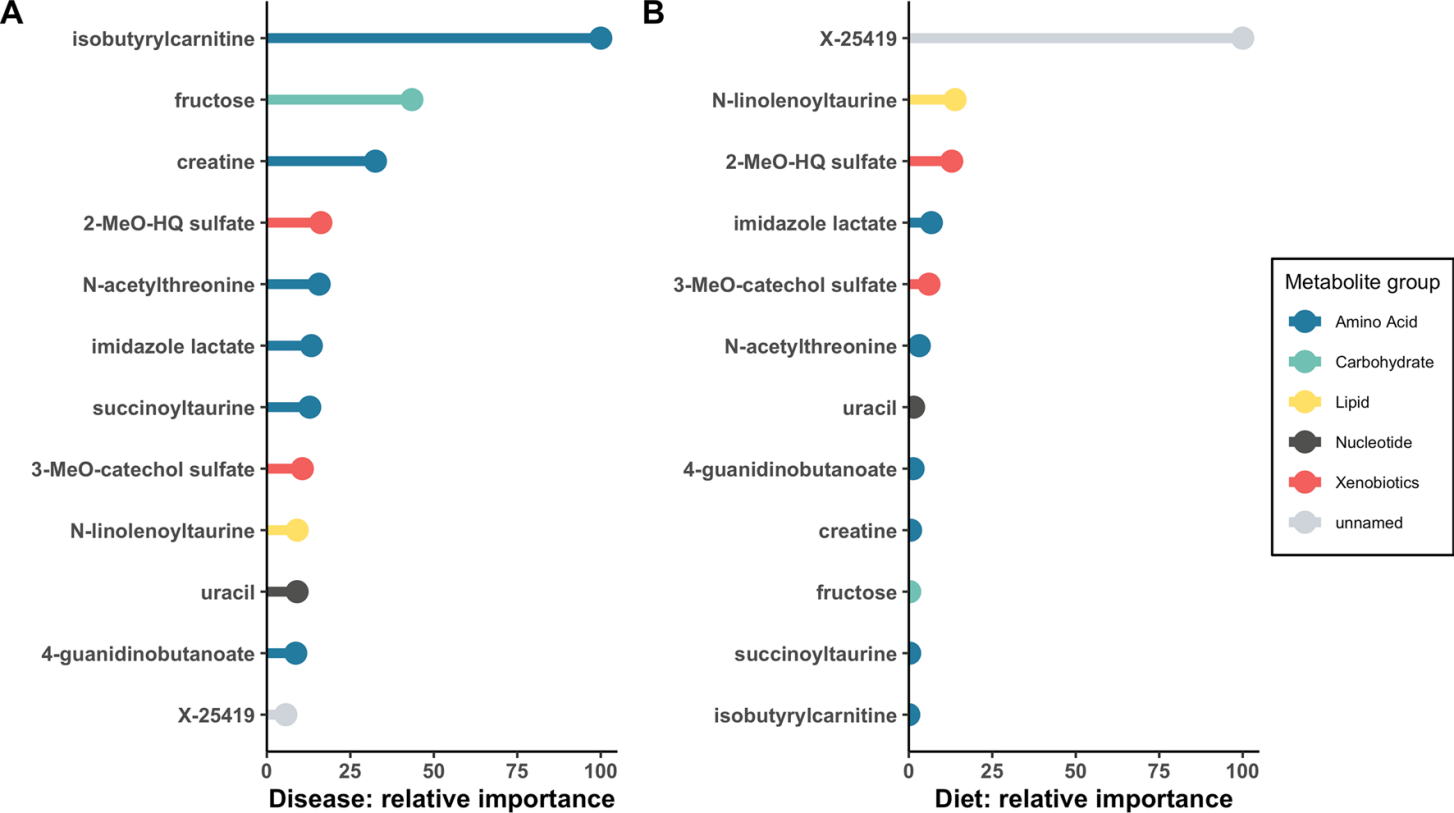
Random forest machine learning analysis was used to evaluate the 12 metabolites that overlapped between disease and diet comparisons at baseline in 75 dogs for their relative contribution in the biochemical importance plot. The super pathway for each metabolite is indicated by color as defined by the legend on the right. (**A**) Comparison based on disease (DCM vs. healthy controls). These metabolites had a predictive accuracy of 86%, (**B**) Comparison based on diet. These metabolites had a predictive accuracy of 87%. Key: 2-MeO-HQ sulfate, 2-methoxyhydroquinone sulfate (1); 3-MeO-catechol sulfate, 3-methoxycatechol sulfate (2).

In order to assess potential relationships between diet and DCM, we compared the two sets of metabolites that significantly distinguished disease (n = 153) and diet (n = 63), after correction for multiple comparisons. Twelve metabolites were common to both sets (Table 2), with ten being higher in both the dogs with DCM and in dogs eating NT diets. To assess the potential contribution of diet to these 12 metabolites, we compared baseline and post-dietary intervention concentrations in the DCM-NT group (Table 2). Eight of the 12 metabolites changed significantly following the 9-month dietary intervention. Six of the ten metabolites that were higher in the DCM-NT group at baseline decreased significantly between baseline and post-dietary comparisons. To determine whether the 12 shared metabolites were predictive of disease and diet status, we applied a random forest machine learning approach. These 12 shared metabolites had a predictive accuracy of 86% for disease and 87% for diet (Fig. 1A,B, in which the predictive importance of each metabolite relative to the other metabolites is plotted). Isobutyrylcarnitine was the most predictive marker for DCM and X-25419 was the most predictive marker for NT diet. In addition to analyzing the 12 metabolites as individual molecules, we calculated pairwise correlations to examine potential relationships among the 12 compounds (Supplementary Fig. S1). The most highly correlated pair of molecules (r = 0.71, *P* = 3.96E-12) was imidazole lactate and X-25419.

**Table 2:** Metabolites that overlapped in comparisons of metabolites that differed by diet and metabolites that differed by disease in 75 dogs (46 dogs with DCM [non-traditional (NT) diet, n = 38; traditional (T) diet, n = 8] and 29 healthy controls [NT diet, n = 12; T diet, n = 17]).

Chemical name	Pubchem ID	Super pathway	Sub pathway	Disease status			Diet			
				Beta	SE	*P-* value	Beta	SE	*P-* value	Post-intervention change*
*N*-acetylthreonine	152204	Amino acid	Glycine, serine, and threonine metabolism	5.232	1.334	3.17E-07	0.370	0.072	2.20E-06	NS
Imidazole lactate	793	Amino acid	Histidine metabolism	2.831	0.685	7.13E-08	0.761	0.140	6.89E-07	NS
Isobutyrylcarnitine (C4)	168379	Amino acid	Leucine, isoleucine, and valine metabolism	3.397	0.822	8.34E-09	0.585	0.139	7.41E-05	NS
Succinoyltaurine	–	Amino acid	Methionine, cysteine, SAM, and taurine metabolism	3.121	0.864	2.13E-06	0.533	0.128	8.82E-05	NS
Creatine	586	Amino acid	Creatine metabolism	2.669	0.631	1.08E-10	1.001	0.210	9.95E-06	↓
4-guanidinobutanoate	500	Amino acid	Guanidino and acetamido metabolism	2.679	0.712	1.50E-07	0.775	0.172	2.42E-05	↓
Fructose	2723872	Carbohydrate	Fructose, mannose, and galactose metabolism	7.190	1.800	3.71E-09	0.355	0.080	3.06E-05	↓
N-linolenoyltaurine	–	Lipid	Endocannabinoid	2.042	0.569	9.34E-06	0.860	0.147	1.29E-07	↓
Uracil	1174	Nucleotide	Pyrimidine metabolism, uracil- containing	3.563	1.032	1.85E-05	0.406	0.089	1.95E-05	↓
3-methoxycatechol sulfate (2)	–	Xenobiotics	Benzoate metabolism	− 0.890	0.237	1.99E-05	− 1.444	0.291	4.59E-06	↑
2-methoxyhydroquinone sulfate (1)	–	Xenobiotics	Benzoate metabolism	− 1.698	0.430	3.25E-07	− 1.096	0.216	3.04E-06	↑
X-25419	–	Unknown	Unknown	0.770	0.196	3.55E-06	3.102	0.239	2.20E-20	↓

After merging these two datasets, 12 metabolites overlapped both comparisons. Positive beta values indicate higher levels in dogs with DCM (for disease status) and in dogs eating NT diets (for diet status). Of 38 DCM-NT dogs enrolled in the 9-month intervention, 20 were alive for the 9-month post-intervention analysis (See also Supplementary Table S5). Six of the ten metabolites that were higher in the DCM-NT group at baseline decreased significantly between baseline and post-intervention analysis.

Key: *SE* standard error, *SAM* S-adenosyl methionine, *significant change from baseline to 9-month post-intervention analysis after change to intervention diet (↓ significant decrease; ↑ significant increase; *NS* change not significant).

### Gene set and pathway enrichment analysis

We conducted pathway analyses to investigate biological pathways of disease. In order to augment pathway enrichment analysis, we note that metabolites interact with proteins, such as enzymes and transporters, and those proteins can be assessed for enriched bioprocesses and pathways relevant to the significantly different individual metabolites and sets of metabolites. We sought to use this complementary approach to deepen our understanding of the mechanisms contributing to diet-associated DCM. This parallel approach began with protein-metabolite pairs, and used the proteins of significantly enriched protein-metabolite pairs to build protein–protein interaction (PPI) networks. Separate networks were built from the metabolites significantly different based on disease (disease metabolite set) and the metabolites significantly different based on diet (diet metabolite set), resulting in networks of 1127 and 2863 total PPIs, respectively. From these two PPI networks, we selected the 137 PPI pairs shared between the disease and diet networks, yielding 47 unique proteins. Importantly, each PPI pair contains at least one protein that both interacts with two or more metabolites in both the disease metabolite set and the diet metabolite set and reached FDR-corrected significance in the MBROLE results^27^. The 47 genes encoding those proteins (Supplementary Table S2) then underwent gene set enrichment analysis with the MSig C2 dataset of 6290 different gene sets and pathways^28^.

A total of 105 pathways and gene sets passed FDR-corrected *P* values and were represented by two or more genes. As the MSig C2 dataset is a large collection drawn from diverse sources, there are a number of similarly named and overlapping gene sets. In this regard, we observed significant enrichment with eight different nuclear factor kappa B (NFкB) gene sets, with *P* values ranging from 2.48E-04 to 5.24E-45, the latter representing the canonical NFκB pathway from WikiPathways^29^. Other top enriched pathways include keratinization from Reactome (*P* = 1.13E-38)^30^; targets of protein kinase C alpha (PRKCA) and its effector erythroblast transformation specific (ETS1; *P* = 2.63E-22); targets of Kaposi’s sarcoma-associated herpesvirus (KSHV) miR-K12-11 (also known as miR155; *P* = 3.67E-12); glycine, serine, and threonine metabolism from KEGG (*P* = 1.93E-11); endosomal sorting complex required for transport (ESCRT, from Reactome; *P* = 1.93E-11), and downregulated during adipocyte differentiation (*P* = 1.93E-11). Other gene sets and pathways relate to oxidative damage and other inflammatory mediator pathways such as tumor necrosis factor alpha and interleukin-18 (Supplementary Table S3).

## Part 2: Analysis of Dogs with DCM (n = 46) Eating Non-Traditional or Traditional Diets

### Metabolomic signatures

**Figure 2:**
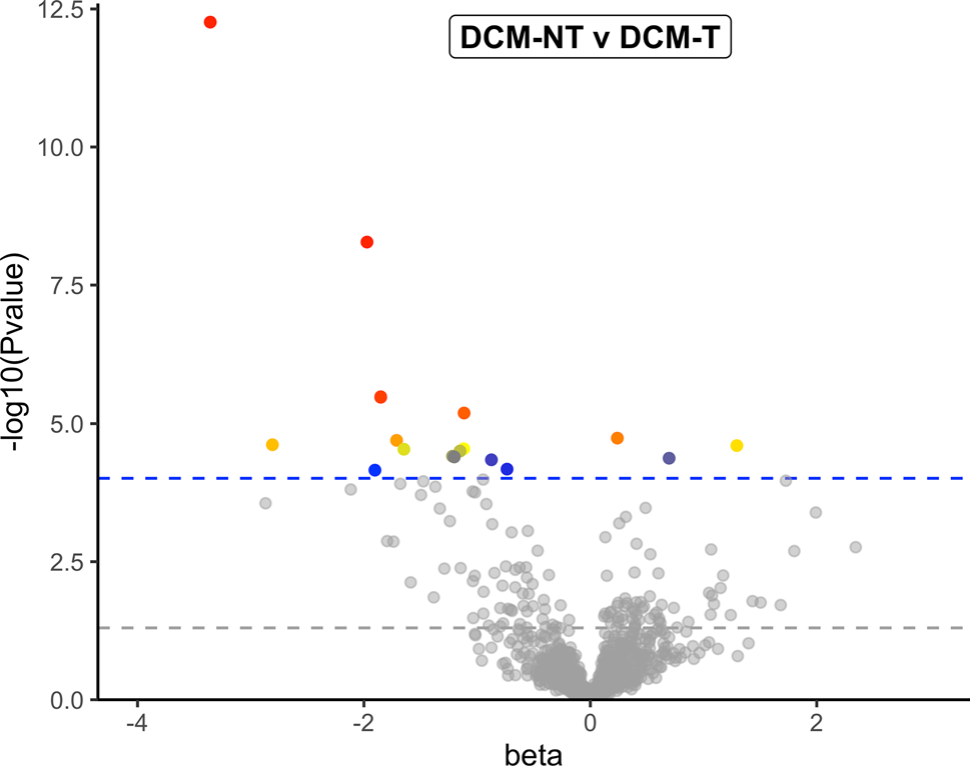
Illustration of metabolites that were significantly different between groups at baseline in 46 dogs with dilated cardiomyopathy (DCM): 38 dogs with DCM eating non-traditional diets (DCM-NT) and 8 dogs with DCM eating traditional diets (DCM-T) at baseline. Metabolites plotted in gray have *P* values above the cutoff of 9.78E-05 and are considered not statistically significantly different between disease groups, whereas metabolites in colors were statistically significant with a *P* value < 9.78E-05. The beta values, where a negative value denotes higher levels in the DCM-NT diet group, are plotted against the negative of log_10_(*P* value).

In order to examine potential contributions of NT diets to DCM (in contrast to dogs with DCM eating T diets which were presumed to have primary DCM), we conducted additional analyses focused exclusively on the 46 dogs with DCM at baseline. A comparison of dogs with DCM according to baseline diet (DCM-NT [n = 38] vs. DCM-T [n = 8]) identified 17 metabolites as significantly different at the FDR level (*P* < 0.0000978) with 140 metabolites being FDR or nominally significant (Supplementary Table S4; Fig. 2). Because this comparison was based on diet, we also conducted “lookups” of metabolites that were FDR or nominally significantly different between DCM-associated and DCM-non-associated diets from our prior foodomics analysis^20^. Only three of the 17 metabolites that were significantly different at the FDR-corrected threshold for dogs eating the two diet types in the current study (DCM-NT vs. DCM-T) were also FDR-level significant in our prior analysis of the diets associated or unassociated with DCM (X-25419, homoarginine, and X-26008)^20^. When we extended the foodomics lookups to the set of 140 nominally significant metabolites that differed in the two dog groups (*P* < 0.05), 33 compounds were FDR or nominally significant in our prior foodomics analyses (Supplementary Table S4)^20^. Among those 33 metabolites were fatty acids (linolenate [alpha or gamma; 18:3n3 or 6] and docosapentaenoate [n3 DPA; 22:5n3], and 12,13-dihydroxy-9-octadecenoic acid [12,13-DiHOME]); biomarkers of legume intake (tryptophan betaine, trigonelline); and amino acids or amino acid-derived compounds (e.g., glutamate, 4-guanidinobutanoate, argininate, *N*-methylproline; Supplementary Table S4)^20^.

### Post-dietary intervention metabolite changes in dogs with DCM that ate non-traditional diets at baseline

Having identified 140 metabolites that distinguished the two baseline diets (NT and T) consumed by dogs with DCM at the FDR or nominally significant level, we anticipated that metabolites would change after dogs with DCM that had been eating a NT diet at baseline completed a 9-month dietary intervention of a T diet. In the DCM-NT group, 20 dogs with baseline metabolomic analyses were alive at the 9-month time point and were compared at baseline and post-intervention. Eleven metabolites changed significantly between the baseline and post-intervention time points, with 218 additional metabolite changes being nominally significant (Supplementary Table S5). Among the 11 metabolites that decreased significantly were X-25419, tryptophan betaine, and X-26008, while 3-bromo-5-chloro-2,6-dihydroxybenzoic acid and 3,5-dichloro-2,6-dihydroxybenzoic acid increased significantly. Metabolites that changed at a nominal level of significance include creatine, *N*-linolenoyltaurine, fructose, trigonelline, and homoarginine (all decreased), 3-methoxycatechol sulfate and glycine (increased). Within-group changes for the DCM-T group were not analyzed because only two dogs survived to the 9-month timepoint.

## Part 3: Other Analyses

### Pathway enrichment analysis distinguishing dogs based on disease (n = 75) versus dogs with DCM (n = 46) based on diet

**Figure 3:**
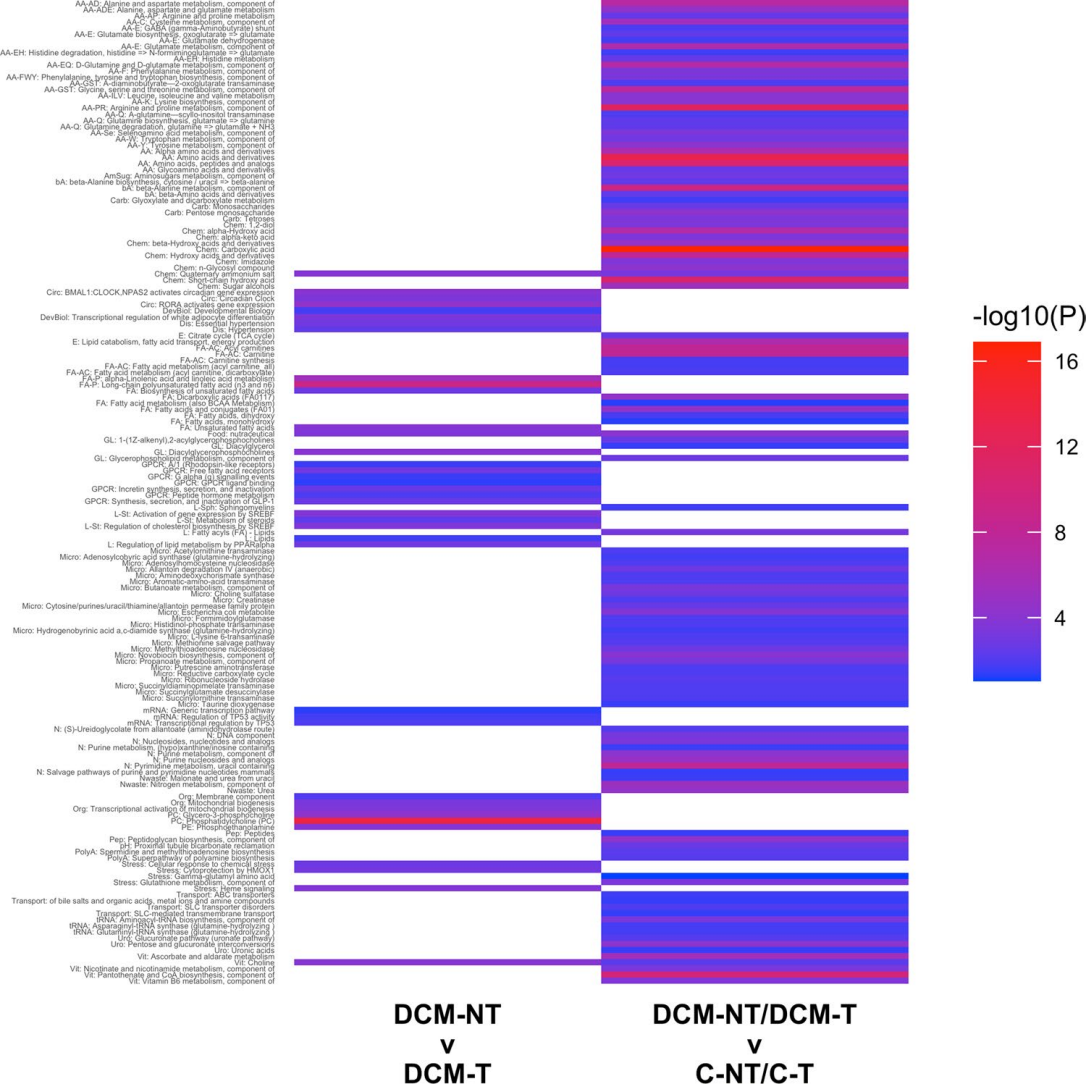
Tile plot showing pathway enrichments for the comparison of dogs by disease (dogs with DCM eating non-traditional diets [DCM-NT] + dogs with DCM eating traditional diets [DCM-T]) vs. (healthy controls eating non-traditional diets [C-NT] + healthy controls eating traditional diets [C-T]) and by diet (DCM-NT vs. DCM-T). Color reflects the corrected *P* value of enrichment, and white indicates the pathway was not significant in that comparison. The pathways that were enriched in the comparison by disease were almost completely different from the pathways enriched in the comparison by diet in dogs with DCM. Of 160 pathways, only three (choline, food nutraceutical, and quaternary ammonium salt) pathways were shared between these two comparisons.

Dogs with diet-associated DCM are clinically different from and have a different response to treatment compared to dogs with primary DCM^12–15,19^, supporting a different etiology of disease (i.e., diet, rather than genetic cause). Therefore, we next sought to understand whether pathways that distinguished (1) dogs with DCM from healthy dogs (regardless of baseline diet) differed from pathways that distinguished (2) dogs with DCM based on diet. These pathways might illuminate causal pathways that are similar or different in diet-associated versus primary (genetic) DCM. Using the sets of 153 FDR-level significant disease-associated metabolites (Supplementary Table S1) and 140 (FDR and nominally significant) diet-associated metabolites (Supplementary Table S4) that distinguished the groups, we performed separate pathway enrichment analyses by disease status ([DCM-NT + DCM-T] vs. [C-NT + C-T]) and dogs with DCM eating different diets (DCM-NT vs. DCM-T). There was very little in common between the pathways enriched in the comparison by disease status and the pathways enriched in the comparison by diet in dogs with DCM (Fig. 3). Only three pathways (choline, food nutraceutical, and quaternary ammonium salt) of 160 were shared between these two comparisons. Among the pathways that differed between healthy controls and dogs with DCM were glutamate and glutamine metabolism; histidine degradation; branched chain amino acids (leucine, isoleucine, valine metabolism), TCA cycle; and acyl carnitine metabolism. In contrast, the pathways that differed by diet type within the group of dogs with DCM (DCM-NT vs. DCM-T) included alpha linolenic acid and linoleic acid metabolism, long-chain polyunsaturated fatty acid (omega-3 and omega-6), regulation of lipid metabolism by peroxisome proliferator-activated receptor alpha, and mitochondrial biogenesis.

### Relationships between metabolites and high-sensitivity cardiac Troponin I (cTnI)

Because serum cTnI is an important biomarker of myocardial damage, we sought to examine its relationship with individual blood metabolites to identify any significant correlations. Correlations between cTnI and individual metabolites were analyzed for all 75 dogs at baseline (Supplementary Table S6). Forty-three metabolites were significantly correlated with cTnI, with an additional 277 metabolites being nominally significant. Among the significantly correlated metabolites were amino acids and proline-containing peptides (e.g., *N,N*-dimethyl-pro-pro, *N,N,N*-trimethyl-alanylproline betaine, imidazole propionate, alpha-ketoglutaramate, cys-gly, oxidized, and guanidinoacetate), acyl carnitines, and plasmalogens. None of the 12 overlapping metabolites from Part 1 were significantly correlated with cTnI, although 4 of 12 metabolites were nominally significant (2-methoxyhydroquinone sulfate (1) [r = −0.303, *P* = 8.82E-03], 3-methoxycatechol sulfate (2) [r = −0.327, *P* = 4.55E-03], creatine [r = 0.410, *P* = 2.73E-04], isobutyrylcarnitine [r = 0.288, *P* = 1.31E-02]). From Part 2, none of the 17 metabolites that were significantly different between the DCM-T and DCM-NT groups at baseline were significantly correlated with cTnI. Examples include homoarginine (r = −0.185, *P* = 1.17E-01); X-25419 (r = −0.034, *P* = 7.78E-01); X-26008 (r = 0.007, *P* = 9.53E-01); and tryptophan betaine (r = 0.005, *P* = 9.67E-01).

## Discussion

Possible causes for diet-associated DCM include a nutrient that is deficient in the diet, causing a deficiency in the dogs, or a dietary compound that is directly or indirectly causing injury to the dogs’ hearts. The latter possibility required looking both at individual metabolites and at enriched pathways. Therefore, the current study applied a multi-faceted metabolomics approach to characterizing diet-associated DCM in dogs and included comparing metabolites and pathway enrichment at baseline, evaluating the impact of a dietary intervention, and assessing overlaps between diet and disease status.

A key finding was metabolic signatures that distinguished dogs with DCM eating NT diets (DCM-NT; presumably diet-associated DCM) from dogs with DCM eating T diets (DCM-T; primary DCM). Most metabolites were higher in dogs with DCM eating NT diets (vs. dogs with DCM eating T diets) and decreased after an intervention diet (which also was associated with clinical and echocardiographic improvement)^15^. These metabolic differences between dogs with DCM eating the two different diet types could reflect different underlying mechanisms for diet-associated DCM compared with primary DCM. However, they also may reflect diet differences because the metabolic signatures of the 46 dogs with DCM separated by diet were similar to those seen in all 75 dogs (DCM and healthy) separated by diet. In addition, 33 of the metabolites that were significantly or nominally significantly different between dogs in the DCM-NT and DCM-T groups matched metabolites that differed between DCM-associated and DCM-non-associated diets in our previous foodomics study (although only three were FDR-level significant on both analyses: homoarginine and two unnamed metabolites)^20^. Many of these nominally different metabolites were amino acids or fatty acids. Metabolites that were higher in both DCM-associated diets^20^ and in the circulation of dogs with DCM eating NT diets in the current study could be considered potential candidates for disease etiology. We might expect candidate metabolites to be associated with a biomarker of myocardial damage (cTnI). However, none of the 33 metabolites that were significantly different both between the DCM-NT and DCM-T groups in the current study and between diets in the previous foodomics study were correlated with cTnI, so it is possible that they might be markers rather than causal compounds. Troponin concentrations may be related more to the amount of myocyte damage rather than to the cause of the damage so the lack of correlation does not prove or disprove a possible role for these compounds. Alternatively, it is possible that a compound in the diet might be metabolized by dogs and therefore, one of the metabolites detected in the blood might be the direct result of the dietary compound after metabolism.

We also sought to characterize mechanisms of diet-associated DCM by identifying metabolites that were shared by both the diet status and disease status comparisons. The set of 12 metabolites that overlapped the disease-based comparison (DCM-NT + DCM-T vs. C-NT + C-T) and the diet-based comparison (DCM-NT + C-NT vs. DCM-T + C-T) was a small, but diverse group. Eight of these 12 compounds changed significantly (6 decreased and 2 increased) following the intervention with T food. Random forest machine learning analysis showed that these 12 overlapping metabolites had a strong predictive accuracy for both disease and diet (86% and 87%, respectively), with isobutyrylcarnitine the most predictive marker for DCM and unnamed compound, X-25419, the most predictive marker for NT diet. One compound that changed, 4-guanidinobutanoate, was also nominally different between the two diet types we evaluated in our previous foodomics analysis^20^. Two of the eight compounds (3-methoxycatechol sulfate and 2-methoxyhydroquinone sulfate) were classified as xenobiotic benzoates, and were the only ones of the 12 overlapping metabolites that were lower at baseline in both the disease- and diet-based comparisons, and both increased post-intervention in the DCM-NT group. Another metabolite that changed (decreased) post-intervention, was the amino acid creatine. Phosphocreatine participates in the phosphorylation of ADP to ATP to supply energy for cardiac contraction and creatine was previously reported to be higher in the blood of people with DCM^22^. The unnamed metabolite, X-25419, was significantly higher in both the disease- and diet-based comparisons at baseline and decreased significantly after the 9-month intervention. The findings of higher X-25419 at baseline and significant decreases after a dietary intervention are consistent with results from our prior foodomics study, as well as from a recent study of apparently healthy dogs eating grain-free or grain-inclusive diets at baseline and, in a subset of dogs with subclinical cardiac abnormalities, 12 months after diet change^31^. Surprisingly, in that study, X-25419 levels remained higher after 12 months in dogs with subclinical cardiac abnormalities originally eating grain-free diets compared to those originally eating grain-inclusive diets. In the current study, none of the 12 overlapping metabolites was significantly correlated with cTnI, although four (2-methoxyhydroquinone sulfate, 3-methoxycatechol sulfate, creatine, and isobutyrylcarnitine) were nominally significant. The lack of correlation between the overlapping metabolites is not definitive evidence that they do not have a primary connection. In addition, the consistent results across both the disease and diet comparisons and significant interrelationships do provide valuable clues. The identity and biochemical functions of the unnamed metabolites also could be helpful.

Identification of a simplistic mechanism for diet-associated DCM is appealing (e.g., a nutrient that is lower in the diet results in a nutritional deficiency in the dog or compound in the diet inhibits absorption of a nutrient thereby causing a deficiency). However, we hypothesize more complex relationships, such as a compound that is only harmful when it is in combination with other factors (e.g., acute kidney injury caused by the combination of melamine and cyanuric acid^32^, or toxicity caused by a compound in an animal with a specific genetic predisposition). Other more complex possibilities include a dietary compound that is metabolized by dogs into a harmful byproduct or metabolite, or a dietary compound that activates or downregulates a pathway involved in myocardial health or disease. To evaluate some of these more complex mechanisms that could contribute to diet-associated DCM, we also conducted gene set and pathway enrichment analysis. While unique pathway enrichment results are expected from metabolomic and genetic analyses of DCM or congestive heart failure alone^33,34^, our analysis used PPI pairs shared by disease and diet networks to investigate the interaction between diet and DCM. Some of the 105 significant pathways and gene sets included multiple pathways related to NFкB; keratinization; targets of protein kinase C alpha and ETSI; targets of KSHV miR-K12-11 (microRNA-155); glycine, serine, and threonine metabolism; and endosomal sorting complex required for transport (ESCRT), as well as pathways relating to oxidative damage and other inflammatory mediator pathways such as tumor necrosis factor alpha and interleukin-18. In the current study, inflammation emerges as an important theme from the pathway enrichment/gene set analyses in which NFкB, tumor necrosis factor, and IL-18 were identified as pathways that were significantly enriched.

In addition to inflammation, there are a number of pathways of interest to investigate in more detail. For example, protein kinase C alpha is involved in calcium regulation and an important determinant in cardiac contractility^35^ and microRNA-155 was reported to be lower in people with DCM^36^. Another pathway of interest is the glycine, serine, and threonine metabolism pathway. One of the proteins driving significance of this pathway was GATM, which catalyzes the synthesis of creatine, homoarginine, and 4-guanidinobutanoic acid (see below).

Another approach to evaluate these more complex relationships is to compare pathways that distinguish dogs with DCM of different etiologies. To do this, we performed separate pathway enrichment analyses of metabolites that differed by disease status in all 75 dogs and metabolites that differed in the 46 dogs with DCM that were eating different diets. There were many different pathways that were significant in each of these individual comparisons (Fig. 3), but what was most striking was the absence of overlap between these comparisons which suggests that metabolic pathways, and potentially causal mechanisms, are very different between primary and diet-associated DCM. Among the chemical classes/pathways detected in the DCM group based on diet type but not in the DCM versus healthy control group were those related to polyunsaturated fatty acids (e.g., alpha linolenic and linoleic acid, omega-3 and omega-6 fatty acids), regulation of lipid metabolism by peroxisome proliferator-activated receptor-alpha, multiple G protein-coupled receptors, and mitochondrial biogenesis. These observations are of interest for several reasons. First, the long-chain polyunsaturated fatty acids, alpha linolenic (18:3, omega-3) and linoleic (18:2, omega-6), must be obtained through the diet. Fatty acids are oxidized in the mitochondria as the primary energy source in the healthy heart. In addition, these two 18-chain fatty acids are precursors to arachidonic acid (20:4, omega-6) and eicosapentaenoic acid (20:5, omega-3) which are substrates for pro-inflammatory and anti-inflammatory mediators, respectively^37,38^. In general, enriched pathways that were different between all dogs with DCM vs. healthy controls were amino acid-related whereas enriched pathways in the DCM-NT vs. DCM-T comparison were lipid-related.

As expected (but not previously published), dogs with DCM as a group had a different metabolic signature than healthy dogs. Despite the absence of previous metabolomic studies of DCM in dogs, findings from the current study substantially overlap with those identified for primary DCM in people and rodent models. As in the current study, human studies of nonischemic DCM identified amino acids and derivatives (e.g., glutamic acid, glutamine, 3-methylhistidine) and multiple individual acyl carnitines as distinguishers of individuals with disease from healthy individuals^22,24^. Biological pathways such as the TCA cycle, oxidative stress as reflected in glutathione metabolism, and systemic inflammation have been identified in people with DCM and/or heart failure and in relevant tissue from other species (i.e., heart muscle in a hamster model of DCM)^23,25,39–41^, and were confirmed in the current study. In addition, while there are only a limited number of metabolomics studies in dogs with naturally-occurring heart disease, two studies of dogs with myxomatous mitral valve disease identified overlapping chemical classes (e.g., amino acids and acyl carnitines) and pathways related to congestive heart failure^42,43^. Specific molecules and pathways detected for both canine cardiac diseases include 3-methylhistidine (a marker of myofibrillar degradation), plasmalogens (the most abundant phospholipid in the cardiac sarcoplasmic reticulum)^44^, the TCA cycle and fatty acid/carnitine metabolism. Some metabolites that were significantly different between dogs with DCM and healthy controls were significantly or nominally significantly positively correlated with cTnI (e.g., creatine, *N,N*-dimethyl-pro-pro, glutamate, some acyl carnitines).

Deficiencies of many different nutrients can cause a secondary form of DCM, including taurine (or its precursors, methionine and cystine), carnitine, B vitamins, choline, vitamin E, and selenium^3,16–18^. Therefore, these nutrients were of special interest in comparing our results from dogs with DCM eating the two different diet types. Compared to the T diet group, dogs with DCM in the NT diet group had higher taurine (nominally significant) but there were no significant differences in methionine, cysteine, cystine, carnitine, thiamine, riboflavin, pyridoxal, choline, or tocopherols (alpha, beta, or gamma). These are consistent with results from analysis of circulating levels of many of these nutrients in small numbers of dogs with diet-associated DCM in a recent publication that showed no significant differences between diet groups^15^. Results from a recent paper in laboratory dogs fed diets restricted in methionine and cystine suggested that plasma and whole blood taurine concentrations may not reflect taurine depletion in muscle, warranting additional research^45^. In addition to more classical nutrients, metabolomics analysis may provide insights into dietary factors that might be “deficient” or present in insufficient levels in a diet that could contribute to disease. For example, the appearance of the diverse chemical class “benzoates” as a distinguisher of both disease and diet status may be novel to this DCM study. Most benzoate metabolites are broadly categorized as xenobiotic, meaning they are supplied by foods (usually plants) or generated by gut microbes. Hydroxybenzoic acids, for example, are found in many plants including grains and legumes such as peas^46,47^. The benzoate, 3-methoxycatechol sulfate that was lower in both DCM and NT diet groups (one of the 12 overlapping metabolites) and increased post-intervention shows evidence as a marker of whole grain intake in people^48^. Other benzoates, such as salicylic acid, are found predominantly in plants other than grains including fruits and vegetables. Benzoic acid and salicylic acid are metabolized primarily through conjugation to glycine to yield hippurate and 2-hydroxyhippurate (salicyurate), respectively^49,50^. Hippurate was nominally higher in dogs with DCM and 2-hydroxyhippurate nominally higher in dogs eating NT diet at baseline. Glycine, which can be rate-limiting for benzoic acid conjugation^51^, was nominally lower in dogs with DCM, and increased post-intervention in dogs with DCM that ate NT diets at baseline. Of potential relevance to DCM are studies showing that sodium benzoate administration can alter carnitine metabolism in people and animal models^52^, and benzoic acid analogs have been shown to inhibit carnitine biosynthesis^53^. A definitive role for specific benzoates or benzoates as a chemical class cannot be concluded from our analyses, but in light of the importance of carnitine in fatty acid availability to the heart and the prominence of benzoates in plant-based ingredients, further research into a possible contributory role is warranted.

Based on the current results and our previous foodomics research^20^, we hypothesize that diet-associated DCM is more likely to be caused by excessive levels of a nutrient or compound in the diet, rather than a deficiency. This is consistent with the protracted nature of improvement in dogs clinically affected with this form of DCM (as opposed to dogs with a simple nutrient deficiency that often have a faster and more complete response). One possibility is that high levels of certain dietary compounds interfere with the availability of nutrients that are essential for heart function. For example, while carnitine was not different between diets in our previous foodomics study or between dogs with different forms of DCM in the current study, other compounds that were higher in NT diets such as the D isomer of carnitine, acetyl-D,L carnitine, deoxycarnitine, choline, betaine, and other betainized compounds (e.g., tryptophan betaine) could reduce carnitine bioavailability in the myocardium by inhibiting carnitine uptake through its transporter, SLC22A5^54–56^. Our observation that carnitine derivatives (e.g., isobutyrylcarnitine, arachidoylcarnitine (C20), oleoylcarnitine (C18:1)) are higher in the blood of dogs with DCM compared to healthy dogs supports the possibility that carnitine transport or metabolism are impaired. The additional measurement of small molecule metabolites and carnitine-specific proteins (e.g., carnitine transporters and carnitine-containing enzymes) in the myocardium could increase understanding of potential mechanisms.

We further hypothesize that excessive levels of several other compounds identified in the current study could contribute to DCM through multiple mechanisms. For example, 4-guanidinobutanoate (gamma-guanidinobutyric acid), one of 12 metabolites overlapping both the disease and diet comparisons has been found in a mushroom (*Trogia venenata*) blamed by some for Yunnan sudden unexplained death syndrome, although it occurs in these mushrooms along with a number of other compounds that may be solely or partially responsible for toxic effects^57–59^. Gamma-guanidinobutanoate is synthesized from arginine and gamma-aminobutyric acid via the enzyme arginine: glycine amidinotransferase, encoded by the GATM gene which was one of the 47 genes encoding proteins observed in the shared PPI of the disease- and diet-associated networks and part of the enriched KEGG glycine, serine, and threonine metabolism pathway. This enzyme also is responsible for creatine and homoarginine synthesis^60^. Negative effects of guanidinobutanoate could result from impaired creatine metabolism which can limit ATP in the heart. Beta- and gamma-guanidinobutyric acid are creatine analogs that compete with creatine for uptake into cells through the creatine transporter and deplete myocardial creatine and phosphocreatine (despite higher circulating creatine concentrations)^22,61^. In rats fed a diet containing beta-guanidinobutryic acid, myocardial creatine and phosphocreatinine were depleted, resulting in reduced cardiac contractility^61,62^. Certain fatty acids/lipids also can have toxic effects and warrant further investigation. For example, erucic acid (erucate), which was significantly higher in all dogs eating NT diets compared to all dogs eating T diets, and nominally higher in all dogs with DCM compared to all controls (and in DCM-NT vs. DCM-T), has been associated with myocardial lipidosis, necrosis, and fibrosis^63^. 9,10-Epoxyoctadecenoic acid (also known as leukotoxin or 9,10-EpOME) is derived from linoleic acid and has been shown to have toxic effects on the heart in dogs^64,65^. 9,10-epoxyoctadecaenoic acid was not detected in the current study but 9,10-dihydroxyoctadecenoic acid (9,10-DiHOME), its metabolite, was nominally higher in the DCM-NT vs. DCM-T comparison. Although the prevailing hypotheses for the cause of diet-associated DCM is too much of a dietary factor, the lack of grains and the compounds they provide (e.g., benzoates) could also complement a dietary factor excess. Similarly, a combination of factors (e.g., toxicity plus a nutrient deficiency or genetic polymorphism) also may be necessary.

This study had limitations that are important to address. The sample size was relatively small, especially for dogs with DCM eating T diets. The low percentage of dogs eating T diets is not unexpected as so many dogs with DCM in recent years are eating NT diets (between 64 and 95% in 4 recent studies)^12,14,15,66^, and because grain-free diets have become so popular (43% of all kibble diets in 2019)^67^. However, this limited statistical power for some analyses and prevented analyses of post-intervention changes in the DCM-T group as only two dogs in this group survived to 9 months. The DCM-NT group was comprised of a wide range of breeds that included both breeds predisposed to primary DCM and more atypical breeds. The typical breeds are challenging to categorize because, while some had clinical and echocardiographic improvements and were therefore likely to have had diet-associated DCM, others in this group might have had primary DCM and just happened to be eating a NT diet. Possible misclassification could have skewed the study's results. Another limitation of the study was variable severity of disease. Most dogs with DCM in both the DCM-NT and DCM-T groups had CHF, but the severity varied and a small number of dogs with DCM in both diet groups were asymptomatic. Our study is also limited by its reliance on blood metabolomics and the absence of measurements in the tissue of interest (myocardium). Therefore, additional research is warranted, including metabolomics and proteomics in the myocardium. Lipidomics analysis in both the blood and myocardium may also provide valuable information, as would pathological evaluation of the myocardium and in vitro testing of cardiac myocytes with compounds of interest. Other limitations are diet-related. The definitions used for NT and T diets have varied among studies^12–15,19,68^. The definitions in the current study focused on the presence or absence of ingredients but still might not be optimal because, until the exact cause is known, it is impossible to specifically target ingredients or certain compounds that are lacking or in excess in the food. Therefore, definitions might need further refinement. In addition, metabolomics only measures small metabolites (typically < 1500 Da) which hinders identification of larger dietary compounds. Not all dogs were changed to the same intervention diet for the 9-month study. Although a single diet would have been ideal in terms of study design, it was not medically optimal for the dogs or practical in this clinical study. The many differences in the metabolite profile in dogs eating different diets emphasizes the importance of addressing diet in metabolomic studies in any species. Finally, some metabolites identified in our study may be generated by the gastrointestinal microbiome and this was not evaluated but warrants further research.

The current study applied metabolomics, pathway and functional group enrichment, PPI network analyses, and machine learning approaches to investigate how dogs with primary or diet-associated DCM differed metabolically from healthy controls, and from each other. Despite ongoing research efforts since the first reports of diet-associated DCM, the causal dietary components and mechanisms have remained elusive. Our results strongly support that underlying mechanisms differ between diet-associated and primary DCM. These findings have the potential to identify therapeutic targets to prevent or delay DCM progression, and validate the application of canine DCM as a spontaneous animal model for human DCM.

## Methods

This study was approved by the Cummings School of Veterinary Medicine Clinical Studies Review Committee (006.18) and the University of Florida Institutional Animal Care and Use Committee (201,810,504), and all experiments were performed in accordance with their guidelines and regulations. Owners of the pet dogs enrolled in the study signed an informed consent form at the time of enrollment. Reporting in the manuscript follows the recommendations in the Animal Research: Reporting of In Vivo Experiments (ARRIVE) guidelines.

### Subjects

Pet dogs diagnosed with naturally-occurring DCM were continuously enrolled between September, 2018 and March, 2020 from both study sites. The study’s definition of DCM consisted of an M-mode fractional shortening (FS) ≤ 25%, normalized left ventricular internal diameter in diastole ≥ 1.8, and normalized left ventricular internal diameter in systole ≥ 1.2 (or breed-specific criteria for Doberman pinschers or Boxers)^12,13,15,69,70^. Eligible dogs had to be eating a commercial NT or T extruded (kibble) diet as their main source of calories for at least 6 months. Baseline diets were categorized as NT if they were grain-free or included pulses or potatoes in the top 10 ingredients, and T if they were grain-inclusive and had no pulses or potatoes in the top 10 ingredients^10,15,26^. Ingredients were determined based on the ingredient list of the diet providing the majority of calories to each dog. Oils (e.g., corn oil) were not classified as a grain product.

Dogs were included as healthy controls based on history, physical examination, echocardiogram, complete blood count, and serum biochemistry profile. For the metabolomics analysis, dogs with DCM and healthy controls were required to be eating the NT or T diet up until and including the time of study enrollment and blood collection.

### Procedures

This metabolomics analysis was part of a larger prospective study evaluating clinical signs, echocardiographic measurements, and cardiac biomarkers in pet dogs with DCM eating NT or T diets^15^. Inclusion/exclusion criteria for enrollment, including the definition of DCM, are described in detail in the prospective study^15^. For metabolomics analysis, blood was collected from dogs with DCM and from healthy controls at the time of enrollment (baseline) in an EDTA tube and plasma was separated and frozen at − 80 °C until analysis of all samples at a single time point. Serum high-sensitivity cardiac troponin I (cTnI) was measured at baseline in all dogs (Gastrointestinal Laboratory, Texas A&M College of Veterinary Medicine, College Station, Texas). Medical treatment of DCM was at the discretion of each dog’s primary clinician. In most instances, taurine supplementation was initiated at the baseline visit. Owners were instructed to administer taurine supplementation until laboratory results were available 2–4 weeks later and to continue supplementation if plasma or whole blood taurine concentrations were low or borderline, although no dogs had low plasma or whole blood taurine concentrations^15^. The owners were given the choice to continue or discontinue taurine supplementation if plasma and whole blood taurine concentrations were found to be normal or high.

In addition to medical treatment which was at the discretion of each dog’s primary clinician, owners of dogs with DCM in both diet groups were instructed to change to 1 of 6 commercial extruded diets that were reduced in sodium, grain-inclusive, did not contain pulses or potatoes in the top 10 ingredients, were made by manufacturers that met the World Small Animal Veterinary Association Global Nutrition Committee’s guidelines^71^, and were diets that had been subjectively associated with clinical improvement in dogs with diet-associated DCM in the investigators’ collective experiences. The intervention diet represented a single experimental treatment for dogs in both the NT and T diet groups (i.e., changing the diet from what the dogs had been originally eating at the time of diagnosis to an intervention diet that had the properties noted above). Within the intervention diet, diet options had variable caloric densities, manufacturers, and costs to address different dog and owner needs. In some dogs with concurrent medical conditions, a different diet was selected to tailor the diet to the individual dog’s needs (e.g., higher fiber, lower fat). All dogs ate primarily an extruded diet but 3 canned options were available to supplement the extruded diet if desired by the owner or if dogs would not eat extruded food alone. Investigators were not blinded to the original diet nor to the intervention diet.

Metabolomics analysis was repeated in dogs in the DCM-NT group, if still alive, 9 months after the diet change.

### Metabolomic analysis

Metabolic profiling of EDTA plasma was conducted by a commercial laboratory using standardized methods (Metabolon, Inc., Morrisville, North Carolina, USA). Metabolites were quantified using ultra‐high‐performance liquid chromatography-tandem mass spectroscopy and identified by comparison to a reference library of 4500 purified standards containing retention time, molecular weight, mass-charge ratio, and mass spectroscopy spectral data. A total of 1036 metabolites that met quality control standards were included in the analyses.

### Statistical analyses

Sample sizes for the current study were not calculated as the number of samples analyzed were all samples that met the eligibility criteria for metabolomics analysis from the parent prospective study at the time of the dogs’ diagnosis of DCM^15^. Within-group changes also were compared for all dogs in the DCM-NT group that survived to the end of the 9-month study. Within-group changes for the DCM-T group were not analyzed because only two dogs survived to the 9-month timepoint.

Demographic data were compared between the groups at baseline using Fisher’s Exact tests for categorical variables and Kruskal–Wallis tests for continuous variables. Statistical analyses comparing dogs at baseline were conducted in SPSS version 28 (SPSS 28.0, IBM Corp., Armonk, New York, USA), with a *P* value < 0.05 considered statistically significant.

## Part 1

### Metabolomic signatures

Prior to analyses, metabolites were batch-normalized, imputed, and natural log-transformed according to standard Metabolon procedures to achieve normal distributions. Linear regression was used to assess associations between groups of dogs according to disease status (DCM or healthy) and diet consumed at the time of enrollment (NT or T) and the individual metabolites measured in plasma. Baseline metabolites were compared between the following groups: (1) Dogs with DCM eating NT diets (DCM-NT) + dogs with DCM eating T diets (DCM-T)] were compared to healthy control dogs eating NT diets (C-NT) + healthy control dogs eating T diets (C-T) and (2) Dogs eating NT diets (DCM-NT + C-NT) were compared to dogs eating T diets (DCM-T + C-T). For analyses of metabolites, a false discovery rate (FDR) statistical significance threshold for metabolites was defined by constructing a correlation matrix in the metabolome dataset to establish independent metabolites^72^. Based on 511 independent metabolites, the significance threshold was calculated as 0.05/511 = 0.0000978. *P* values < 0.05 and ≥ 0.0000978 were considered to be nominally significant. Analyses for individual metabolites were conducted in SAS 9.4 (SAS Institute, Cary, North Carolina, USA).

Links were assessed between the diet and DCM by comparing metabolites at baseline that differed by disease status (DCM-NT + DCM-T) vs. (C-NT + C-T) or by diet status (DCM-NT + C-NT) vs. (DCM-T + C-T) using linear regression models while controlling for age and sex. The two sets of significant metabolites were compared to identify those that overlapped (i.e., were significant from the perspectives of both disease status and diet status). We used linear regression models to assess these overlapping metabolites in the DCM-NT group for changes between baseline and post-intervention and to determine whether their changes were in the expected direction. Machine learning random forest approaches were applied to examine how accurately the overlapping metabolites predicted diet and disease status.

For the 12 metabolites identified by random forest as predictive of disease, Pearson correlation analyses, done in R with the cor function, identified relationships in all dogs combined (i.e., dogs with DCM and healthy dogs, n = 75). These results were assessed with corrplot in R, with hierarchical clustering (hclust) set to 3 with the ward.D2 method.

### Gene set and pathway enrichment analysis

Sets of metabolites that were significantly different between comparison groups, or those that distinguished one group from another were assessed for pathway and functional group enrichment with the MBROLE^27^ and Reactome^30^ platforms, using default settings, and with metabolite classifications from Metabolon. Enrichment was considered only for those pathways and functional groups represented by two or more metabolites from the diet or disease comparison groups. MBROLE and Reactome return FDR-corrected enrichments. To assess enrichment of Metabolon classifications, we calculated a Z-score^73^, which was converted to a* P* value with the pnorm function in R: 2 × pnorm(-abs(Z)). Adjustment of initial *P* values by FDR was done with p.adjust in R^74^.

### Protein–protein interaction networks

Results from MBROLE derived from analysis of blood metabolites identified as significantly different between dogs in different disease groups ([DCM-NT + DCM-T] vs. [C-NT + C-T]) and diet groups ([DCM-NT + C-NT] vs. [DCM-T + C-T]), include significantly enriched protein-metabolite interactions. We used these protein-metabolite interactions, filtered for those protein-metabolite interactions represented by two or more metabolites with an FDR-corrected *P* value to build PPI networks and more broadly assess the functional implications of each set of significantly different metabolites. Networks were built with the Human Reference Interactome (HuRI) tool^75^ using the proteins from the significant protein-metabolite interactions and default settings. All input proteins plus those protein interactions identified by HuRI underwent gene set enrichment analysis using the C2 data (chemical and genetic perturbations, and canonical pathways) of 6290 gene sets from the MSigDB collection^28^ augmented by a recent release (January, 2022) of WikiPathways^29^. Enrichment was determined by Z-score and converted *P* value with FDR correction, as described above^73,74^.

## Part 2

Linear regression was used to assess associations between dogs with DCM at baseline based on their diets at the time of enrollment (DCM-NT vs. DCM-T) and the individual metabolites measured in plasma. For these analyses of metabolites, an FDR statistical significance threshold for metabolites was defined as in Part 1 above. Then, pertinent to this comparison, overlap with biochemical compounds previously found to be significantly different in a study of diets associated versus not associated with DCM were noted^20^.

Paired analyses of baseline and post-intervention metabolites were conducted in the DCM-T and DCM-NT groups to evaluate the hypothesis that the difference between baseline and post-intervention metabolite concentrations was equal to 0. Baseline to post-intervention changes were not performed for the DCM-T group since only two dogs in this group survived to the 9-month re-evaluation time point.

## Part 3

Pathways significantly enriched by the 3 methods described in section “*Gene Set and Pathway Enrichment Analysis*” for the DCM-NT vs DCM-T and the DCM-NT + DCM-T vs C-NT + C-T comparisons were plotted with ggplot in R. Pathways were grouped by common function and the corrected *P* values were used to indicate strength of enrichment on a blue-red spectrum to create a tile plot (Fig. 3).

Pearson correlation analyses were used to examine relationships between metabolites and cTnI at baseline in all dogs combined (i.e., dogs with DCM and healthy dogs, n = 75).

## Supplementary Information

Supplementary Information 1.

Supplementary Information 2.

Supplementary Information 3.

Supplementary Information 4.

Supplementary Information 5.

Supplementary Information 6.

Supplementary Information 7.

## Data Availability

The datasets generated during and/or analyzed during the current study are not publicly available due to ongoing research but are available from the corresponding author on reasonable request.

## References

[1] Stern JA, Ueda Y (2019). Inherited cardiomyopathies in veterinary medicine. Pflugers Arch..

[2] Haas J (2015). Atlas of the clinical genetics of human dilated cardiomyopathy. Eur. Heart J..

[3] Van Vleet JF, Ferrans VJ (1986). Myocardial diseases of animals. Am. J. Pathol..

[4] Marinescu V, McCullough PA (2011). Nutritional and micronutrient determinants of idiopathic dilated cardiomyopathy: Diagnostic and therapeutic implications. Expert Rev. Cardiovasc. Ther..

[5] Sidney S (2019). Association between aging of the US population and heart disease mortality from 2011 to 2017. JAMA Cardiol..

[6] Morita H, Komuro I (2018). Heart failure as an aging-related phenotype. Int. Heart J..

[7] Li H (2020). Targeting age-related pathways in heart failure. Circ. Res..

[8] United States Food and Drug Administration. FDA investigating potential connection between diet and cases of canine heart disease https://wayback.archive-it.org/7993/20201222194256/https://www.fda.gov/animal-veterinary/cvm-updates/fda-investigating-potential-connection-between-diet-and-cases-canine-heart-disease (2018).

[9] United States Food and Drug Administration. FDA investigation into potential link between certain diets and canine dilated cardiomyopathy - February 2019 update https://www.fda.gov/animal-veterinary/news-events/fda-investigation-potential-link-between-certain-diets-and-canine-dilated-cardiomyopathy-february (2019).

[10] United States Food and Drug Administration. FDA investigation into potential link between certain diets and canine dilated cardiomyopathy https://www.fda.gov/animal-veterinary/outbreaks-and-advisories/fda-investigation-potential-link-between-certain-diets-and-canine-dilated-cardiomyopathy (2019).

[11] Jones, J., Carey, L. & Palmer, L. A. FDA update on dilated cardiomyoipathy: Fully and partially recovered cases. *Scientific Forum Exploring Causes of Dilated Cardiomyopathy in Dogs* https://www.ksvdl.org/resources/dilated-cardiomyopathy-dogs-forum.html (2020).

[12] Adin D (2019). Echocardiographic phenotype of canine dilated cardiomyopathy differs based on diet type. J. Vet. Cardiol..

[13] Freid KJ (2021). Retrospective study of dilated cardiomyopathy in dogs. J. Vet. Intern. Med..

[14] Walker AL (2021). Association of diet with clinical outcomes in dogs with dilated cardiomyopathy and congestive heart failure. J. Vet. Cardiol..

[15] Freeman L (2022). Prospective study of dilated cardiomyopathy in dogs eating nontraditional or traditional diets and in dogs with subclinical cardiac abnormalities. J. Vet. Intern. Med..

[16] Fascetti AJ, Reed JR, Rogers QR, Backus RC (2003). Taurine deficiency in dogs with dilated cardiomyopathy: 12 cases (1997–2001). J. Am. Vet. Med. Assoc..

[17] Keene BW (1991). Myocardial L-carnitine deficiency in a family of dogs with dilated cardiomyopathy. J. Am. Vet. Med. Assoc..

[18] Reeves WC, Marcuard SP, Willis SE, Movahed A (1989). Reversible cardiomyopathy due to selenium deficiency. J. Parenter. Enteral Nutr..

[19] Kaplan JL (2018). Taurine deficiency and dilated cardiomyopathy in golden retrievers fed commercial diets. PLoS ONE.

[20] Smith CE, Parnell LD, Lai C-Q, Rush JE, Freeman LM (2021). Investigation of diets associated with dilated cardiomyopathy in dogs using foodomics analysis. Sci. Rep..

[21] Isserlin R (2010). Pathway analysis of dilated cardiomyopathy using global proteomic profiling and enrichment maps. Proteomics.

[22] Alexander D, Lombardi R, Rodriguez G, Mitchell MM, Marian AJ (2011). Metabolomic distinction and insights into the pathogenesis of human primary dilated cardiomyopathy. Eur. J. Clin. Invest..

[23] Maekawa K (2013). Global metabolomic analysis of heart tissue in a hamster model for dilated cardiomyopathy. J. Mol. Cell. Cardiol..

[24] Verdonschot JAJ (2020). Metabolic profiling associates with disease severity in nonischemic dilated cardiomyopathy. J. Card. Fail..

[25] Haas J (2021). Energy metabolites as biomarkers in ischemic and dilated cardiomyopathy. Int. J. Mol. Sci..

[26] Adin D (2021). Effect of type of diet on blood and plasma taurine concentrations, cardiac biomarkers, and echocardiograms in 4 dog breeds. J. Vet. Intern. Med..

[27] López-Ibáñez J, Pazos F, Chagoyen M (2016). MBROLE 2.0-functional enrichment of chemical compounds. Nucl. Acids Res..

[28] Subramanian A (2005). Gene set enrichment analysis: A knowledge-based approach for interpreting genome-wide expression profiles. Proc. Natl. Acad. Sci. USA.

[29] Martens M (2020). WikiPathways: Connecting communities. Nucl. Acids Res..

[30] Gillespie M (2022). The reactome pathway knowledgebase 2022. Nucl. Acids Res..

[31] Adin, D. B., Haimovitz, D., Freeman, L. M., & Rush, J. E. Untargeted global metabolomic profiling of healthy dogs grouped on the basis of grain inclusivity of their diet and of dogs with subclinical cardiac abnormalities that underwent a diet change. *Am. J. Vet. Res.* **83**(9). 10.2460/ajvr.22.03.0054 (2022). 10.2460/ajvr.22.03.0054 35895762

[32] Puschner B, Reimschuessel R (2011). Toxicosis caused by melamine and cyanuric acid in dogs and cats: uncovering the mystery and subsequent global implications. Clin. Lab. Med..

[33] Tahir UA (2021). Metabolomic profiles and heart failure risk in Black adults: Insights from the Jackson Heart Study. Circ. Heart Fail..

[34] Liu Z (2022). Bioinformatics prediction of potential mechanisms and biomarkers underlying dilated cardiomyopathy. World J. Cardiol..

[35] Singh RM, Cummings E, Pantos C, Singh J (2017). Protein kinase C and cardiac dysfunction: A review. Heart Fail. Rev..

[36] Satoh M, Minami Y, Takahashi Y, Tabuchi T, Nakamura M (2011). A cellular microRNA, let-7i, is a novel biomarker for clinical outcome in patients with dilated cardiomyopathy. J. Card. Fail..

[37] Tortosa-Caparrós E, Navas-Carrillo D, Marín F, Orenes-Piñero E (2017). Anti-inflammatory effects of omega 3 and omega 6 polyunsaturated fatty acids in cardiovascular disease and metabolic syndrome. Crit. Rev. Food Sci. Nutr..

[38] Innes JK, Calder PC (2018). Omega-6 fatty acids and inflammation. Prostaglandins Leukot. Essent. Fatty Acids.

[39] Wong SC, Fukuchi M, Melnyk P, Rodger I, Giaid A (1998). Induction of cyclooxygenase-2 and activation of nuclear factor-kappaB in myocardium of patients with congestive heart failure. Circulation.

[40] Hakuno D, Hamba Y, Toya T, Adachi T (2015). Plasma amino acid profiling identifies specific amino acid associations with cardiovascular function in patients with systolic heart failure. PLoS ONE.

[41] Nemec Svete A (2021). Inflammation and its association with oxidative stress in dogs with heart failure. BMC Vet. Res..

[42] Li Q (2015). Veterinary medicine and multi-omics research for future nutrition targets: Metabolomics and transcriptomics of the common degenerative mitral valve disease in dogs. OMICS.

[43] Li Q (2021). Metabolomics analysis reveals deranged energy metabolism and amino acid metabolic reprogramming in dogs with myxomatous mitral valve disease. J. Am. Heart Assoc..

[44] Gross RW (1985). Identification of plasmalogen as the major phospholipid constituent of cardiac sarcoplasmic reticulum. Biochemistry.

[45] Torres CL, Backus RC, Fascetti AJ, Rogers QR (2003). Taurine status in normal dogs fed a commercial diet associated with taurine deficiency and dilated cardiomyopathy. J. Anim. Physiol. Anim. Nutr..

[46] Kandil A, Li J, Vasanthan T, Bressler DC (2012). Phenolic acids in some cereal grains and their inhibitory effect on starch liquefaction and saccharification. J. Agric. Food Chem..

[47] Magalhães SCQ (2017). European marketable grain legume seeds: Further insight into phenolic compounds profiles. Food Chem..

[48] Zhu Y, Wang P, Sha W, Sang S (2016). Urinary biomarkers of whole grain wheat intake identified by non-targeted and targeted metabolomics approaches. Sci. Rep..

[49] Devries A, Alexander B (1948). Studies on amino acid metabolism. III. Plasma glycine concentration and hippuric acid formation following the ingestion of benzoate. J. Clin. Invest..

[50] Nelson E, Hanano M, Levy G (1966). Comparative pharmacokinetics of salicylate elimination in man and rats. J. Pharmacol. Exp. Ther..

[51] Beliveau GP, Brusilow SW (1987). Glycine availability limits maximum hippurate synthesis in growing rats. J. Nutr..

[52] Sakuma T (1991). Alteration of urinary carnitine profile induced by benzoate administration. Arch. Dis. Child..

[53] Debreceni B, Farkas V, Fischer GM, Sandor A (2005). Effect of aromatic ring-containing drugs on carnitine biosynthesis in rats with special regard to p-aminomethylbenzoic acid. Metabolism.

[54] Tamai I (1998). Molecular and functional identification of sodium ion-dependent, high affinity human carnitine transporter OCTN2. J. Biol. Chem..

[55] Wu X (1999). Functional characteristics and tissue distribution pattern of organic cation transporter 2 (OCTN2), an organic cation/carnitine transporter. J. Pharmacol. Exp. Ther..

[56] Grube M (2011). Selective regulation of cardiac organic cation transporter novel type 2 (OCTN2) in dilated cardiomyopathy. Am. J. Pathol..

[57] Zhou Z-Y (2012). Evidence for the natural toxins from the mushroom *Trogia venenata* as a cause of sudden unexpected death in Yunnan Province, China. Angew. Chem. Int. Ed. Engl..

[58] Stone R (2012). Heart-stopping revelation about how Chinese mushroom kills. Science.

[59] Xu YC, Xie XX, Zhou ZY, Feng T, Liu JK (2018). A new monoterpene from the poisonous mushroom *Trogia venenata*, which has caused Sudden Unexpected Death in Yunnan Province, China. Nat. Prod. Res..

[60] Faller KME (2018). Impaired cardiac contractile function in arginine:glycine amidinotransferase knockout mice devoid of creatine is rescued by homoarginine but not creatine. Cardiovasc. Res..

[61] Lygate CA, Neubauer S, Lopaschuk GD, Dhalla NS (2014). The myocardial creatine kinase system in the normal, ischemic and failing heart. Cardiac energy metabolism in health and disease.

[62] Zweier JL, Jacobus WE, Korecky B, Brandejs-Barry Y (1991). Bioenergetic consequences of cardiac phosphocreatine depletion induced by creatine analogue feeding. J. Biol. Chem..

[63] Yamashiro S, Clandinin MT (1980). Myocardial ultrastructure of rats fed high and low erucic acid rapeseed oils. Exp. Molec. Pathol..

[64] Sugiyama S, Hayakawa M, Nagai S, Ajioka M, Ozawa T (1987). Leukotoxin, 9, 10-epoxy-12-octadecenoate, causes cardiac failure in dogs. Life Sci..

[65] Fukushima A (1988). Cardiovascular effects of leukotoxin (9, 10-epoxy-12-octadecenoate) and free fatty acids in dogs. Cardiovasc. Res..

[66] Freid KJ (2020). Retrospective investigation of diet and dilated cardiomyopathy (DCM) in dogs (abstract). J. Vet. Intern. Med..

[67] Quest BW, Leach SB, Garimella S, Konie A, Clark SD (2022). Incidence of canine dilated cardiomyopathy diagnosed at referral institutes and grain-free pet food store sales: A retrospective survey. Front. Anim. Sci..

[68] Ontiveros ES (2020). Development of plasma and whole blood taurine reference ranges and identification of dietary features associated with taurine deficiency and dilated cardiomyopathy in golden retrievers: A prospective, observational study. PLoS ONE.

[69] Meurs KM (2013). Association of dilated cardiomyopathy with the striatin mutation genotype in boxer dogs. J. Vet. Intern. Med..

[70] Wess G, Domenech O, Dukes-McEwan J, Häggström J, Gordon S (2017). European society of veterinary cardiology screening guidelines for dilated cardiomyopathy in Doberman Pinschers. J. Vet. Cardiol..

[71] World Small Animal Veterinary Association Global Nutrition Committee. Guidelines on selecting pet foods https://wsava.org/wp-content/uploads/2021/04/Selecting-a-pet-food-for-your-pet-updated-2021_WSAVA-Global-Nutrition-Toolkit.pdf (2021).

[72] Li J, Ji L (2005). Adjusting multiple testing in multilocus analyses using the eigenvalues of a correlation matrix. Heredity (Edinb.).

[73] Mangano KM (2021). Diet-derived fruit and vegetable metabolites show sex-specific inverse relationships to osteoporosis status. Bone.

[74] RDocumentation. p.adjust: Adjust P-values for multiple comparisons https://www.rdocumentation.org/packages/stats/versions/3.6.2/topics/p.adjust (2022).

[75] Luck K (2020). A reference map of the human binary protein interactome. Nature.

