# Supplementary material for: Metabolomic profiling in dogs with dilated cardiomyopathy eating non-traditional or traditional diets and in healthy controls
Source: Sci Rep. 2022 Dec 30;12:22585. doi: 10.1038/s41598-022-26322-8 (PMC9803641; doi:10.1038/s41598-022-26322-8)

**Supplementary Figure S1:** Correlation plot of 12 metabolites identified as accurately predicting diet and disease status.

Correlations between pairs of metabolites in the set of 12 metabolites shared by dogs from the disease-based comparison (dogs with dilated cardiomyopathy eating non-traditional or traditional diets vs. healthy control dogs eating non-traditional or traditional diets) and the diet-based comparison (dogs with dilated cardiomyopathy and healthy control dogs eating non-traditional diets vs. dogs with dilated cardiomyopathy and healthy controls eating traditional diets) are plotted by color and with correlation coefficients displayed. Hierarchical clustering of the 12 overlapping metabolites and their relationships to one another identified three clusters, and dotted lines demarcate these clusters. Pearson correlation coefficients between metabolite pairs that pass the threshold of significance (*P*=0.01667) are displayed at the intersection of those two metabolites. Blank squares on the plot indicate non-significant metabolite correlations. Metabolite abbreviations: 2-MeO-HQ sulfate, 2-methoxyhydroquinone sulfate; 3-MeO-catechol sulfate, 3-methoxycatechol sulfate.


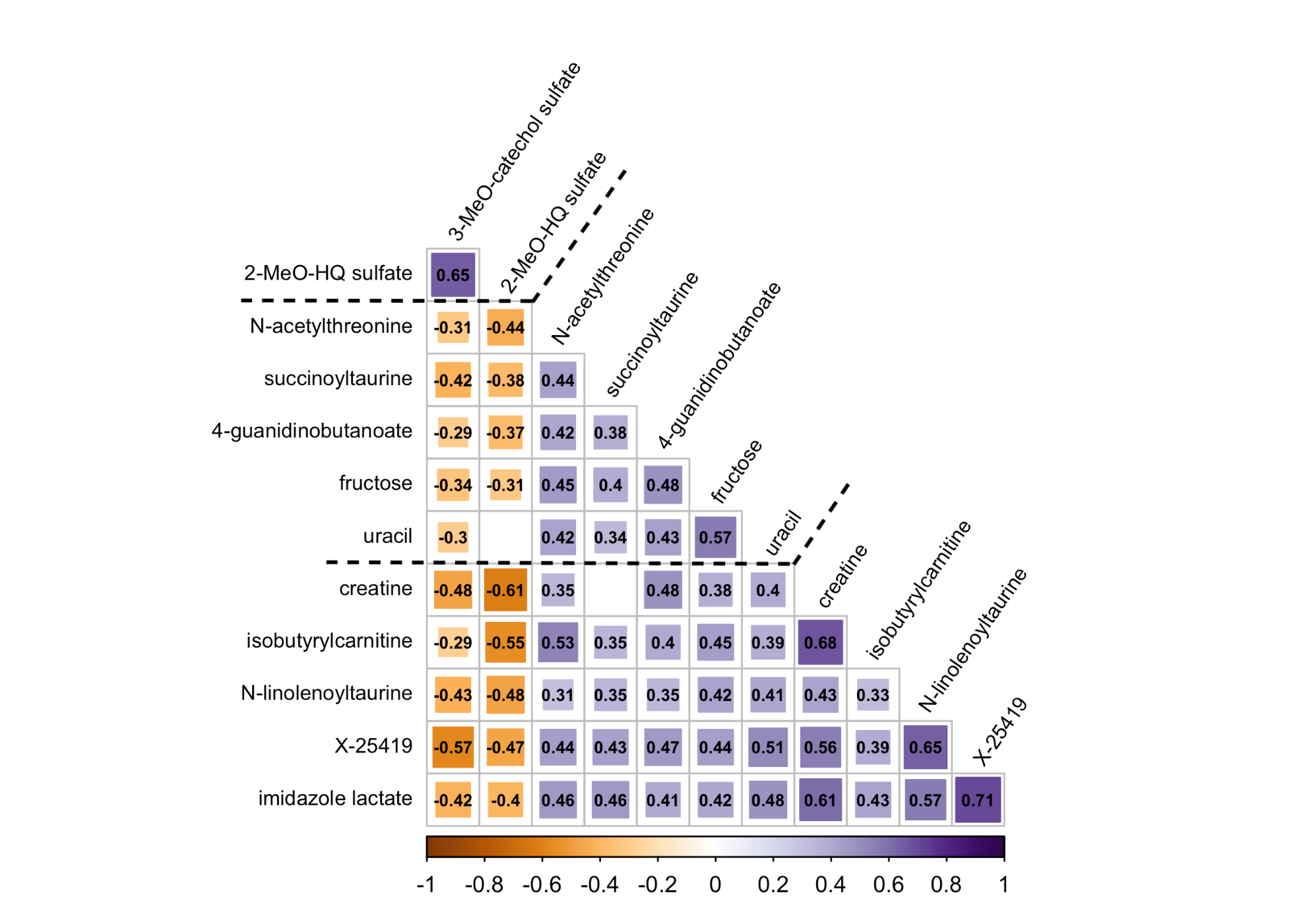

Supplement: Supplementary file 1 — Supplementary Information 1. [file 41598_2022_26322_MOESM1_ESM.docx]
